# Rationale and design of a randomised trial of intravenous iron in patients with heart failure

**DOI:** 10.1136/heartjnl-2022-321304

**Published:** 2022-08-10

**Authors:** Paul R Kalra, John GF Cleland, Mark C Petrie, Fozia Z Ahmed, Paul WX Foley, Philip A Kalra, Ninian N Lang, Rebecca E Lane, Iain C Macdougall, Pierpaolo Pellicori, Michael T B Pope, Michele Robertson, Iain B Squire, Elizabeth A Thomson, Ian Ford

**Affiliations:** 1 Department of Cardiology, Portsmouth Hospitals University NHS Trust, Portsmouth, UK; 2 School of Cardiovascular and Metabolic Health, University of Glasgow, Glasgow, UK; 3 Golden Jubilee National Hospital, Clydebank, UK; 4 Division of Cardiovascular Sciences, The University of Manchester Faculty of Biology Medicine and Health, Manchester, UK; 5 Great Western Hospital, Swindon, UK; 6 Department of Renal Medicine, Salford Royal Hospital, Northern Care Alliance NHS Foundation Trust, Salford, UK; 7 Part of Guy's and St Thomas' NHS Foundation Trust, Royal Brompton and Harefield Hospitals, London, UK; 8 Department of Renal Medicine, King's College Hospital, London, UK; 9 Robertson Centre for Biostatistics, University of Glasgow, Glasgow, UK; 10 Department of Cardiology, University Hospital Southampton NHS Foundation Trust, Southampton, UK; 11 Department of Cardiovascular Sciences, University of Leicester, Leicester, UK; 12 NIHR Leicester Biomedical Research Centre Cardiovascular Diseases, Leicester, UK

**Keywords:** Heart Failure, Systolic, Heart Failure

## Abstract

**Objectives:**

For patients with a reduced left ventricular ejection fraction (LVEF) heart failure with reduced ejection fraction (HFrEF) and iron deficiency, administration of intravenous iron improves symptoms, exercise capacity and may in the following 12 months, reduce hospitalisations for heart failure. The Effectiveness of *I*nt*r*avenous ir*on* treat*m*ent versus standard care in p*a*tie*n*ts with heart failure and iron deficiency (IRONMAN) trial evaluated whether the benefits of intravenous iron persist in the longer term and impact on morbidity and mortality.

**Methods:**

IRONMAN is a prospective, randomised, open-label, blinded endpoint (PROBE) event-driven trial. Patients aged ≥18 years with HFrEF (LVEF ≤45%) and evidence of iron deficiency (ferritin <100 µg/L and/or TSAT <20%) were enrolled if they had either a current or recent hospitalisation for heart failure or elevated plasma concentrations of a natriuretic peptide. Participants were randomised to receive, or not to receive, intravenous ferric derisomaltose in addition to guideline-recommended therapy for HFrEF. Every 4 months, intravenous iron was administered if either ferritin was <100 µg/L or, provided ferritin was ≤400 µg/L, TSAT was <25%. The primary endpoint is a composite of total hospitalisations for heart failure and cardiovascular death. Hospitalisation and deaths due to infection are safety endpoints.

**Results:**

Trial recruitment was completed across 70 UK hospital sites in October 2021. Participants were followed until the end of March 2022. We plan to report the results by November 2022.

**Conclusions:**

IRONMAN will determine whether repeated doses of intravenous ferric derisomaltose are beneficial and safe for the long-term treatment of a broad range of patients with HFrEF and iron deficiency.

**Trial registration number:**

NCT02642562.

**WHAT IS ALREADY KNOWN ON THIS TOPIC**

- In patients with heart failure and reduced ejection fraction and associated iron deficiency, treatment with intravenous iron can improve symptoms, exercise capacity and may reduce hospitalisation for heart failure, in the following 12 months.

**WHAT THIS STUDY ADDS**

- Effectiveness of *I*nt*r*avenous ir*on* treat*m*ent versus standard care in p*a*tie*n*ts with heart failure and iron deficiency (IRONMAN) is a randomised trial, powered to determine whether repeated doses of intravenous iron (ferric derisomaltose) are beneficial and safe in the long term in a broad range of patients with heart failure and reduced ejection fraction. The primary endpoint is a composite of total hospitalisations for heart failure and cardiovascular death.

**HOW THIS STUDY MIGHT AFFECT RESEARCH, PRACTICE OR POLICY**

- The results of IRONMAN will help to inform clinical practice and international guidelines with respect to the management of iron deficiency in patients with heart failure and reduced ejection fraction.

## Introduction

Iron is an essential component of haemoglobin, myoglobin, the mitochondrial electron transport chain and many enzymes. Patients with heart failure, with or without reduced left ventricular ejection fraction (LVEF), often have anaemia, and this will usually be due to iron deficiency.^1–3^ However, iron deficiency is also common in patients without anaemia.^1–3^ Iron deficiency, with or without anaemia, is associated with more severe symptoms and a worse prognosis.^1–3^

Placebo-controlled studies have demonstrated that intravenous administration of ferric carboxymaltose in ambulatory patients with heart failure and reduced LVEF (HFrEF) improves exercise capacity, symptoms and quality of life (QoL).^4 5^ Iron deficiency was defined as serum ferritin <100 µg/L, or between 100 and 300 µg/L if transferrin saturation (TSAT) <20%. In both studies, the respective primary endpoint was evaluated at 24 weeks. A trial of predischarge intravenous ferric carboxymaltose to patients who had been hospitalised with acute heart failure (AFFIRM-AHF)) suggested that treatment to 6 months might reduce the risk of recurrent hospitalisations for heart failure but not cardiovascular mortality when assessed at 52 weeks, although the effect on its primary endpoint, a composite of recurrent hospitalisations for heart failure and cardiovascular death, was of borderline significance.^6^ A meta-analysis of seven randomised trials of patients with HFrEF and iron deficiency found that intravenous iron reduced the risk of hospitalisation for heart failure but was unable to demonstrate a reduction in cardiovascular mortality.^7^

Major gaps in our knowledge remain, including the long-term benefits and safety of repeated administration of intravenous iron. Although the rationale for benefit is clear, bypassing evolutionary systems that have evolved to prevent iron overload also poses theoretical risks, including increased oxidative stress, mitochondrial damage and infection.^8^ These theoretical risks should be confirmed or refuted by clinical evidence. The Effectiveness of *I*nt*r*avenous ir*on* treat*m*ent versus standard care in p*a*tie*n*ts with heart failure and iron deficiency (IRONMAN) trial was designed and conducted in the UK to investigate the benefits and safety of repeated doses of intravenous ferric derisomaltose over an extended period, which should help inform clinical practice and international guidelines.

## Trial design

IRONMAN is a prospective, randomised open-label, blinded endpoint (PROBE) event-driven trial designed to assess the efficacy and safety of intravenous ferric derisomaltose in symptomatic patients with HFrEF and iron deficiency. The endpoints committee adjudicating events are kept blinded to assigned treatment.

Patients aged ≥18 years with new or established symptomatic HFrEF (LVEF ≤45% within the preceding 24 months) were invited to participate. Iron deficiency was defined as serum ferritin <100 µg/L and/or TSAT <20%. In addition, patients either had to have a current or recent (<6 months) admission for heart failure (including daycare intravenous diuretics) or have increased plasma concentrations of a natriuretic peptide (NT-proBNP >250 ng/L in sinus rhythm or >1000 ng/L in atrial fibrillation or equivalent for BNP, box 1).

Box 1 **Inclusion/exclusion criteria**

**Inclusion criteria**

Age ≥18 years

Left ventricular ejection fraction (LVEF) ≤45% within the last 2 years using any conventional imaging modality (most recent assessment)

NYHA class II–IV

Iron deficient – defined as transferrin saturation (TSAT) <20% and/or ferritin <100 ug/L.

Evidence of being in a higher risk heart failure group:

1. Current or recent (within 6 months) hospitalisation for heart failure.
2. Outpatients with NT-proBNP >250 ng/L in sinus rhythm or >1000 ng/L in atrial fibrillation (or BNP >75 pg/mL or 300 pg/mL, respectively).

Able and willing to provide informed consent.

**Exclusion criteria**

Haemoglobin <9 g/dL or 13 g/dL in women or >14g/dL in men.

Ferritin >400ug/L.

eGFR <15 mL/min/1.73 m^2^ (MDRD/CKD-EPI).

Already planned to receive intravenous iron.

Likely to need or already receiving erythropoiesis-stimulating agents.

Blood transfusion in the previous 3 months or active clinically relevant bleeding in the investigator’s opinion or known or suspected gastrointestinal malignancy.

Planned cardiac surgery or revascularisation.

Any major vascular event in the previous 3 months, including type 1 myocardial infarction, cerebrovascular accident, major cardiovascular surgery or percutaneous coronary intervention.

Awaiting or treated by cardiac transplantation or left ventricular assist device.

Active infection (if the patient has significant ongoing infection, recruitment should be postponed until it has resolved or been controlled).

Any disease other than heart failure with a life expectancy of <2 years.

Pregnancy, breast feeding or childbearing potential in the absence of effective contraception.

Contraindication to intravenous iron according to contemporary Summary of Product Characteristics including hypersensitivity to Monofer ® or any of its excipients; known serious hypersensitivity to other parenteral iron products; anaemia due to causes other than iron deficiency (eg, haemolytic anaemia); iron overload or disturbances in utilisation of iron (eg, haemochromatosis and haemosiderosis); and decompensated liver disease.

Participation in another intervention study involving a drug or device within the past 90 days (coenrolment in observational studies is permitted).

BNP, B-type natriuretic peptide; CKD-EPI, Chronic Kidney Disease Epidemiology Collaboration; eGFR, estimated glomerular filtration rate; LVEF, left ventricular ejection fraction; MDRD, modification of diet in renal disease; NYHA, New York Heart Association; NT-proBNP, N-terminal pro B-type natriuretic peptide.

Patients were excluded if they had high ferritin (>400 µg/L), a haemoglobin <9.0 g/dL or an estimated glomerular filtration rate (eGFR) <15 mL/min/1.73 m^2^. Iron deficiency is less common when haemoglobin is more than 1 g/dL above the WHO definition of anaemia. Accordingly, to reduce screen failures, men with a haemoglobin >14 g/dL and women with values >13 g/dL were excluded. A detailed list of inclusion and exclusion criteria is shown in box 1.

## Trial plan

**Figure 1:**
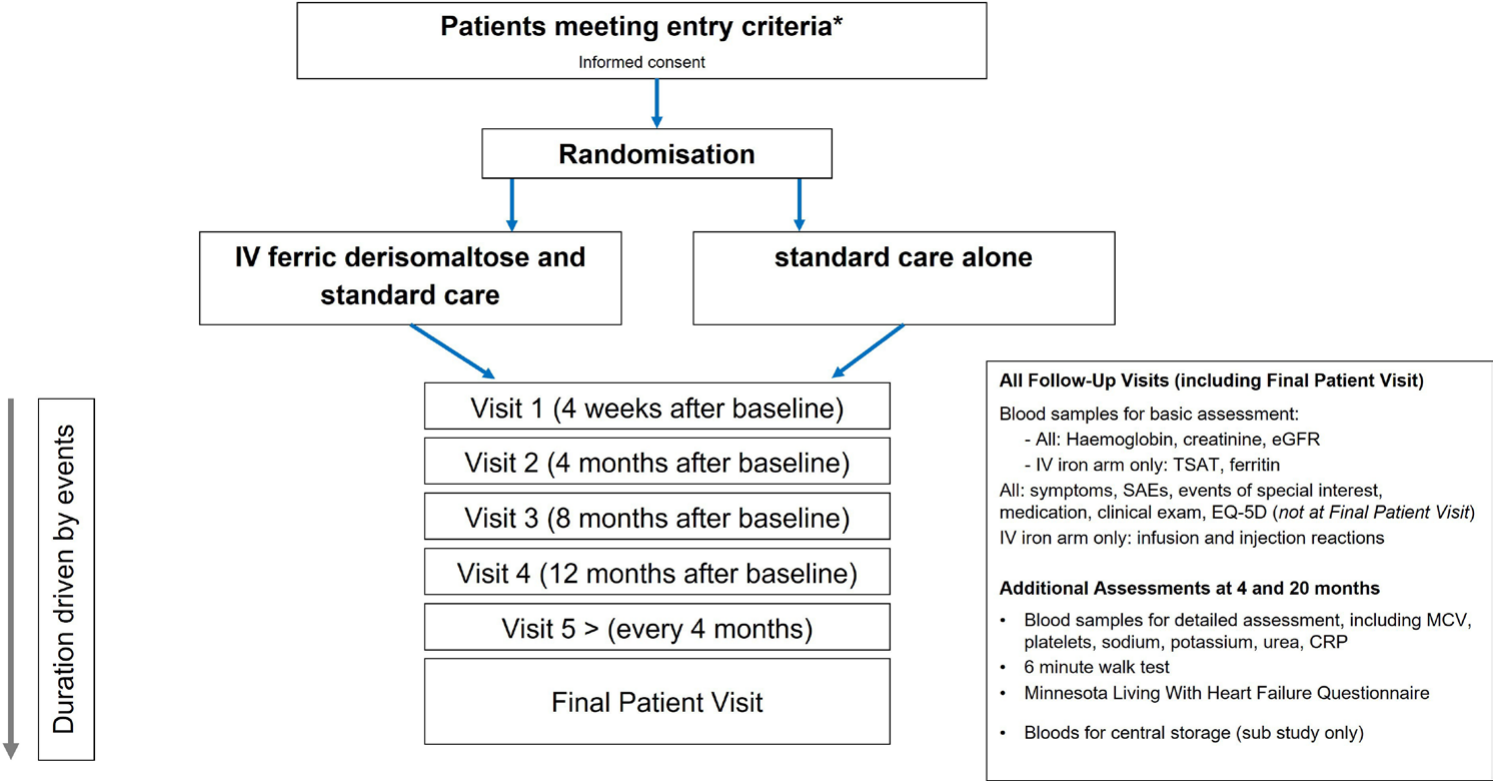
Overview of the Ironman trial: visit schedule and procedures. *See box 1 for full list of inclusion and exclusion criteria. CRP, C reactive protein; EGFR, estimated glomerular filtration rate; SAE, serious adverse event.

An overview of the trial is shown in figure 1 and schedule of assessments in the online online supplemental appendix 1. Written informed consent was obtained for participation in the trial, with additional optional consent for follow-up of electronic medical records (from 1 year prior to consent until 10 years after trial completion) and to provide blood samples at baseline, 4 and 20 months for subsequent biomarker analysis.

10.1136/heartjnl-2022-321304.supp1 Supplementary data

### Randomisation

Consenting and eligible patients were randomised with equal probability to the two treatment arms, stratifying by recruitment context (in hospital, recently discharged or ambulatory with elevated natriuretic peptide) and trial site, using a web-based system based on randomised, permuted blocks of variable size.

### Treatment

Participants assigned to intravenous iron were administered ferric derisomaltose by infusion at a dose determined by haemoglobin value and body weight (table 1). At each visit, investigators were encouraged to optimise heart failure therapy for both groups, according to contemporary guidelines.

**Table 1:** Intravenous ferric derisomaltose infusion dose regimen

Haemoglobin	Body weight <50 kg*	Body weight 50– <70 kg	Body weight ≥70 kg
≥10 g/dL	20 mg/kg	1000 mg	20 mg/kg up to a maximum of 1500 mg
<10 g/dL	20 mg/kg	20 mg/kg	20 mg/kg up to a maximum of 2000 mg

The dosing table permits an estimation of iron need and utilises a pragmatic approach that takes into consideration a patient’s weight and haemoglobin concentration. Doses up to and including 1000 mg – infused over >15 min. Doses exceeding 1000 mg – infused over 30 min or more.

*At trial design guidance for calculating iron need for this patient group was limited; therefore, a pragmatic administration approach was taken to ensure patients did not receive higher than the maximum licenced dose.

A key goal was to try and ensure that those assigned to intravenous iron were kept iron replete, with redosing with intravenous ferric derisomaltose at the 4-week review and every 4 months after randomisation if either the ferritin was <100 µg/L or, provided ferritin was ≤400 µg/L, TSAT was <25%. If iron deficiency recurred, investigators were encouraged to consider further investigations for potential sources of blood loss. Oral iron supplementation was permitted at the discretion of the investigator in the standard care arm. Any use of non-trial iron supplements was recorded.

The data collected at each timepoint varied, with more in-depth assessment taking place at randomisation, 4 and 20 months (figure 1, online supplemental appendix 1). This included detailed laboratory analysis (local laboratories), 6 min walk test (where possible) and assessments of QoL (Minnesota Living With Heart Failure and EQ-5D).

### Primary and secondary efficacy endpoints

The primary and secondary efficacy endpoints are summarised in box 2. The primary endpoint consists of total hospitalisations for heart failure (first and recurrent) and cardiovascular death. Hospitalisations for heart failure include events where heart failure was the primary or a major contributory reason for admission (with a minimum of an overnight stay). For instance, a patient admitted with a primary diagnosis of atrial fibrillation or myocardial ischaemia who had, at the time of admission, worsening breathlessness and/or increasing peripheral oedema requiring treatment with a loop diuretic, would count as a primary endpoint. To reduce double counting of events, cardiovascular death during a hospitalisation for heart failure and readmissions for heart failure occurring on the same day as discharge from a previous heart failure admission are not counted as recurrent events.

Box 2 **Primary and secondary endpoints**

**Primary endpoint**

Cardiovascular (CV) mortality or hospitalisation for worsening heart failure (analysis will include recurrent hospitalisations)

**Secondary endpoints**

**Secondary efficacy**

1. Hospitalisation for worsening heart failure (recurrent events).
2. CV hospitalisation (first event)
3. CV death or hospitalisation for heart failure analysed as time to first event.
4. Overall Score from Minnesota Living with Heart Failure at 4 months.
5. Cardiovascular mortality.
6. Overall EQ-5D visual analogue score (VAS) at 4 months.
7. Overall EQ-5D index at 4 months.
8. CV mortality or hospitalisation (first event) for major CV event including: stroke, myocardial infarction and heart failure.
9. All-cause mortality.
10. All-cause hospitalisation (first event).
11. Combined all-cause mortality or first all-cause unplanned hospitalisation.
12. Physical domain of Minnesota Living With Heart Failure at 4 months.
13. Physical domain of Minnesota Living With Heart Failure at 20 months.
14. Overall EQ-5D VAS at 20 months.
15. Overall EQ-5D index at 20 months.
16. Overall Score from Minnesota Living with Heart Failure at 20 months.
17. Days dead or hospitalised at 3 years.
18. Quality-adjusted days alive and out of hospital at 12 months.
19. Six minute walk test at 4 months.
20. Six minute walk test at 20 months.

**Secondary safety**

1. Death due to infection.
2. Hospitalisation primarily for infection.

### Safety assessments

Investigators are asked to report serious adverse events (SAEs). This does not include routine treatment or monitoring of heart failure; elective or preplanned treatment for a pre-existing non-cardiac condition; any admission for general care without deterioration in health; and treatment on an emergency, outpatient basis for an event not fulfilling the definition of an SAE.

All emergency day-case treatments for heart failure or elective percutaneous coronary intervention or cardiac device insertion are to be recorded as SAEs. Investigators are expected to report all blood transfusions and any important bleeding event, even if it does not require hospitalisation or was not life threatening. Death due to infection and hospitalisation due to infection are secondary safety endpoints.

Record linkage to national databases of deaths, hospital admissions and incident cancers is planned at the end of the trial in England and Scotland and for deaths in Wales to ensure complete reporting of events.

### Sample size and statistical analysis

Sample size calculations based on recurrent event analyses are difficult (additional sample size calculations are given in online supplemental appendix 2). Conservatively, we based our calculations on a time to first event analysis in a Cox proportional hazards model. We expected to recruit half the participants during a hospitalisation. The anticipated first primary endpoint rate in the control group was 60% at 3 years. We estimated that 570 participants per group (yielding 631 first primary outcomes) would provide 80% power to detect an HR of 0.8 at the 5% significance level. Allowing for non-cardiovascular mortality and some withdrawals of consent for follow-up, we intended to recruit 650 patients per group.

10.1136/heartjnl-2022-321304.supp2 Supplementary data

The Independent Data Monitoring Committee (IDMC) conducted interim analyses of the primary endpoint when approximately 50% and 70% of the target number of first primary endpoints had been reached, requiring p<0.001 to recommend early stopping.

The primary endpoint will be analysed by the method of Lin *et al* 9 including the randomised treatment group and recruitment context as covariates. The estimated rate ratio, 95% CI and p value will be reported, with accumulated events displayed using the method of Ghosh and Lin.^10^

Secondary endpoints will be analysed hierarchically in the order shown in box 2, if the primary analysis is significant at the 5% level. Endpoints in the list will continue to be tested until one fails to reach 5% significance. Power calculations have been carried out for the first four secondary endpoints (online supplemental appendix 2).

Secondary endpoints involving recurrent events will be analysed as for the primary endpoint. Time to first event outcomes will be analysed using Cox proportional hazards models including treatment effect and recruitment context, with the treatment effect HR and 95% CIs estimated with associated p values using the Wald statistic and treatment groups compared graphically using cumulative incidence functions.

QoL scores and 6 min walk tests results at 4 and 20 months will be compared between randomised treatment groups using analysis of covariance, with treatment group and stratification variable as covariates.

Subgroup analyses will be carried out for the primary endpoint, analysed as a recurrent event and then separately as time-to-first event.

A formal Statistical Analysis Plan will be finalised before trial database lock.

### Modifications to sample size

In practice, we recruited mainly outpatients who had a lower rate of events; recruitment was slower than expected, especially during the COVID-19 pandemic, which also may have reduced cardiovascular admissions, resulting in a lower event rate for the primary endpoint. This, in combination with likely patient and investigator fatigue, led the Trial Steering Committee (TSC) to revise the power calculation for the trial. Assuming an HR of 0.75, as in AFFIRM-AHF trial,^11^ we calculated that 379 first primary endpoints would now provide 80% power at the 5% significance level.

Analyses based on patients randomised until the end of March 2020 with a censoring date of 30 September 2020 will be carried out to assess the impact of the COVID-19 pandemic on the results.

### Trial oversight and management

National Health Service Greater Glasgow and Clyde and the University of Glasgow are cosponsors for the trial, supported by the Glasgow Clinical Trials Unit with trial data and statistical centre in the Robertson Centre for Biostatistics, University of Glasgow.

Four committees were established to oversee the trial delivery (memberships detailed in online supplemental appendix 3): TSC, IDMC, Trial Management Group and the Clinical Endpoint Committee. An overview of committee roles is provided in box 3.

10.1136/heartjnl-2022-321304.supp3 Supplementary data

Box 3 **Committee roles and responsibilities**

The trial is cosponsored by National Health Service (NHS) Greater Glasgow and Clyde and the University of Glasgow. Four committees have been established to oversee the trial delivery (membership of these committees is detailed in online supplemental appendix 3).

- Trial Steering Committee (TSC): the TSC includes an independent chairperson, at least two other independent members, a representative from the British Heart Foundation and a patient representative. The TSC provides overall supervision of the trial, ensuring it is conducted in accordance with the principles of Good Clinical Practice (GCP) and the relevant regulations. The TSC is responsible for approving the trial protocol and any protocol amendments.
- Independent Data Monitoring Committee (IDMC): the IDMC consists of three independent experts (a cardiologist, a renal physician and a biostatistician) and is responsible for overseeing trial conduct, conducting interim analyses and monitoring patient safety.
- Clinical Endpoint Committee (CEC): the CEC carry out blinded assessment of all clinical events identified as potentially relevant to the designated secondary health outcomes. This included blinded adjudication of all hospitalisations and deaths.
- Trial Management Group (TMG): the TMG comprises the chief investigator, other coapplicants, project manager and sponsor representatives and meet frequently to monitor all aspects of trial conduct and progress and to ensure protocol adherence.

### Patient and public involvement

Patients were involved at all stages of trial design, including membership of the TSC.

### Results

Participants were enrolled in UK centres from August 2016 to October 2021 (list of investigators in online supplemental appendix 4). Follow-up will be completed by April 2022. It is anticipated that the results will be presented by November 2022.

10.1136/heartjnl-2022-321304.supp4 Supplementary data

## Discussion

The IRONMAN trial will provide important information on the benefits and safety of intravenous ferric derisomaltose, in patients with HFrEF and iron deficiency. IRONMAN has important differences from previous studies of intravenous iron in heart failure. IRONMAN recruited a broad range of patients from outpatients and admissions including some with new-onset heart failure. There were relatively few exclusion criteria. Anticipating that we would recruit older people with other medical problems and that many participants’ circumstances would change during follow-up, the protocol permitted follow-up by telephone and/or participants’ medical records, if they were unable or unwilling to attend in person. We expected that most participants would consent to record linkage thereby ensuring complete ascertainment of clinical outcomes. These aspects of the trial design have helped maintain data collection throughout the challenges of the COVID-19 pandemic. If the trial indicates benefit, then a health economic analysis will be conducted.

### Definition of iron deficiency

There remain uncertainties regarding the best readily available blood test to identify iron deficiency in patients with chronic disease, including heart failure. Previous studies^4–6^ defined iron deficiency as a ferritin <100 µg/L or, if ferritin was between 100 and 300 µg/L, a TSAT <20%. IRONMAN has a slightly broader definition (ferritin <100 µg/L or TSAT <20% provided ferritin is ≤400 µg/L). Iron homeostasis in patients with chronic disease is complex. For patients with chronic heart failure, inflammation rather than iron deficiency may be a key determinant of serum ferritin. Moreover, inflammatory signalling leading to an increase in hepcidin may reduce iron absorption in the gut, rendering oral iron supplements ineffective.^12^ The important clinical attribute of markers of iron deficiency is their ability to predict a therapeutic response. Iron deficiency is common in patients with heart failure and lower haemoglobin, and it may make little difference which marker is used. Many patients with heart failure will fulfil all the different proposed definitions for iron deficiency.^3^

### Iron preparation and dosing

Published trials^4–6^ evaluated relatively low doses of intravenous iron with repeated administration when insufficient iron was given initially or when iron deficiency recurred (according to the same definition). In CONFIRM-HF (placebo controlled trial evaluating change in 6 min walk test between baseline and 24 weeks), after initial correction of iron deficiency, maintenance treatment was given at a limited dose of 500 mg intravenous ferric carboxymaltose at weeks 12, 24 and 36 if iron deficiency was present.^5^ AFFIRM-AHF used a dosing regimen of 500–1000 mg of intravenous ferric carboxymaltose in the repletion phase (weeks 0 and 6) and, if iron deficiency persisted, 500 mg at weeks 12 and 24.^6^ If iron deficiency adversely affects well-being and prognosis, which can be improved by correcting the deficiency, then it makes sense to ensure that patients are kept iron replete rather than awaiting the recurrence of iron deficiency before intervening. To maintain iron repletion in IRONMAN patients were assessed every 4 months and, in contrast to other trials, redosed if TSAT was <25% (rather than 20%) provided serum ferritin was ≤400 µg/L, or if ferritin was <100 µg/L.

IRONMAN is the first large trial in heart failure to investigate ferric derisomaltose, which can be given as a rapid, high-dose infusion (up to 20 mg/kg). Total dose replenishment was given whenever possible. From a healthcare provider (and patient) perspective, correction of iron deficiency with a single high-dose infusion is attractive. The trial will provide data on longer term iron requirement for patients receiving optimal guideline-directed treatment.

While oral iron was permissible in the standard of care arm, IRONMAN is not designed to determine if intravenous iron is better than oral iron.

### Trial duration

Although AFFIRM-AHF and IRONMAN enrolled similar numbers of patients, follow-up was for 12 months in AFFIRM-AHF and will be considerably longer in IRONMAN, up to 5.6 years. It is plausible that the relatively short follow-up in AFFIRM-AHF impacted on the finding that there was no obvious effect of intravenous iron on cardiovascular death (occurred in 77 patients administered ferric carboxymaltose and in 78 assigned to placebo: HR 0.94 (0.68–1.29); p=0.69).^6^

Although blinding is normally an important part of trial design, there are occasions where it is very difficult to implement. Iron infusions are dark brown, and there are few placebo alternatives. Ensuring a patient does not see what is going into their arm is difficult. It is even harder to blind the investigator, who should not be aware of the haemoglobin, blood tests for iron deficiency or what the participant will receive. This requires blinded and unblinded teams at every centre. Feedback from research teams and patient representatives was that this was unrealistic. As such, IRONMAN incorporated a PROBE design. For studies where the primary endpoint is QoL or exercise capacity, blinding is essential. Outcomes such as heart failure hospitalisation and cardiovascular death are less prone to bias when adjudicated blindly by an independent committee, as in IRONMAN.

### Other considerations

Inevitably, COVID-19 will have influenced the IRONMAN trial. Some participants will have had COVID-19 infections, and some will have died from COVID-19. Visits to research clinics were curtailed during the pandemic, and therefore, assessing patients for recurrent iron deficiency and redosing with iron was impossible at times. There has been a reduction in hospitalisations for heart failure in the UK, as in many other countries, during the pandemic.^13^ Many hospitals introduced ambulatory care for heart failure to try to reduce the need for admission. We will include a COVID-19 sensitivity analysis including all patients randomised until the start of the first UK lockdown (end of March 2020). Clinical experience suggests most patients do not need frequent redosing with intravenous iron once fully replete. Accordingly, we assume that most patients assigned to intravenous iron remained iron replete until 30 September 2020, which will be used as the censoring date for the COVID-19 sensitivity analysis.

The primary endpoint in IRONMAN includes recurrent hospitalisation for heart failure. It has been proposed that this is the most clinically relevant endpoint for patients with heart failure, capturing the total impact of treatment.^14^ It was hoped that this would increase statistical power. However, recent randomised trials have raised uncertainty about the benefit of recurrent event analysis on statistical power.^15 16^

### Safety of intravenous iron

There are theoretical risks associated with repeated intravenous iron dosing. Labile (free) iron can result in the generation of reactive oxygen species, which could lead onto oxidative stress and cell damage.^17^ A meta-analysis of studies across a broad range of conditions found that intravenous iron was associated with an increased risk of infection (rate ratio (RR) 1.17; 95% CI 1.04 to 1.31).^8^ This was not confirmed across heart failure studies. Most studies did not define infection a priori, and the authors note potential bias. A trial investigating liberal versus conservative dosing with intravenous iron sucrose in haemodialysis patients found no difference in infection rates.^18^ IRONMAN, with death and hospitalisation due to infection as safety endpoints, will help clarify the long-term safety of intravenous iron in patients with heart failure.

Two other ongoing randomised outcome trials of intravenous ferric carboxymaltose include patients with HFrEF: HEART-FID^19^ and FAIR-HF 2 (NCT03036462). Important differences (see online supplemental appendix 5) in trial designs should help establish which patients with heart failure get the most benefit from intravenous iron and inform the current disparity among international guidelines.^20–22^

10.1136/heartjnl-2022-321304.supp5 Supplementary data

## Conclusion

IRONMAN will help clarify the long-term efficacy and safety of intravenous ferric derisomaltose in a broad range of patients with HFrEF.

## Data Availability

Data sharing not applicable as no datasets generated and/or analysed for this study.
