# Supplementary material for: Rationale and design of a randomised trial of intravenous iron in patients with heart failure
Source: Heart. 2022 Aug 10;108(24):1979–85. doi: 10.1136/heartjnl-2022-321304 (PMC9726969; doi:10.1136/heartjnl-2022-321304)
Supplement: Supplementary data [file heartjnl-2022-321304supp001.pdf]

Appendix 1: Schedule of Assessments

All visits should be performed within +/- 2 weeks of the documented visit time (e.g. 4 months +/- 2 weeks)

	Screening	Randomisation/ First Infusion	Visit 1	Visit 2	Visit 3	Visit 4	Visit 5	Visit 6	Visits 7 >	Final patient visit
Time from inclusion	*For hospitalised participants, these visits will be close together prior to discharge.*<br><br>*For all participants, screening and randomisation must be completed using blood tests within 6 weeks of the respective visit.*<br><br>*First infusion may be administered up to 7 days post-randomisation.*		4 weeks	4 months	8 months	12 months	16 months	20 months	24 months and then 4-monthly until notified to schedule the final patient visit	*To be completed at participant's scheduled Final patient visit. Visit window to be notified by the CTU. LPLV is expected to be approximately 5.5 years from first randomisation.*
			Bloods will be collected either during the study visit or in advance of visit (within 2 weeks) as part of standard clinical practice. Results must be available prior to any dosing visit.	*Bloods will be collected either during the study visit or in advance of visit (within 3 weeks) as part of standard clinical practice, apart from blood for storage, which will be collected at the visit. Results must be available prior to any dosing visit.*<br><br>*As the study is event driven, the final patient visit cannot be pre-specified.*						
Consent	X									
Demographics	X									
Medical history	X									
Medications (baseline)	X									
Medications (concomitant)			X	X	X	X	X	X	X	X
Inclusion/ Exclusion	X	X								
Randomisation		X								
N-BNP	X*									
TSAT	X		X**	X**	X**	X**	X**	X**	X**	X**

Ferritin	X		X**	X**	X**	X**	X**	X**	X**	X**
Creatinine/eGFR	X	X^^	X	X	X	X	X	X	X	X
Haemoglobin	X	X^^	X	X	X	X	X	X	X	X
MCV, MCHC, MCH		X^^		X				X		
RDW^		X^^		X				X		
Platelets		X^^		X				X		
Sodium, potassium, urea		X^^		X				X		
CRP		X^^		X				X		
Bilirubin^		X^^		X				X		
Albumin^		X^^		X				X		
Random glucose^		X^^		X				X		
Bloods for storage (sub study)		X		X				X		
Infusion **		X***	X***	X***	X***	X***	X***	X***	X***	X***
Serious adverse events and events of special interest		X	X	X	X	X	X	X	X	X
Injection reactions		X**	X**	X**	X**	X**	X**	X**	X**	X**
Minnesota questionnaire		X		X				X		
EQ-5D		X	X	X	X	X	X	X	X	
Clinical Assessment	X	X	X	X	X	X	X	X	X	X
6 minute walk test		X		X				X		
ECG^{+}	X									
Pregnancy test^{++}		X^{++}	X^{++}	X^{++}	X^{++}	X^{++}	X^{++}	X^{++}	X^{++}	X^{++}
LVEF assessment$^{\#}$	X									

Notes:

1. X = assessments made as part of standard clinical practice for patients with chronic heart failure
2. X* = outpatients only without admission in last 6 months
3. X** = active treatment arm (iron) only i.e. 50% of recruits
4. ^ = if available
5. ^^ = use values from assessments within 6 weeks of randomisation if available
6. + = unless there are ECG results in the last 4 weeks prior to the visit
7. $^{++}$ = for women of child-bearing potential receiving IMP.

8. *** = infusion will only be given to those patients in the IV iron arm who meet the re-dosing criteria. If bloods tests taken at the study visit, a separate infusion visit within 3 weeks will be required for those who need re-dosing (anticipated approximately every third visit for those in IV iron arm). If blood tests available within the 3 weeks before study visit then re-dosing, if required, can happen at the main study visit.
9. [#] = If required – an assessment can be carried out if not done in prior 2 years, or most recent result does not permit inclusion

Visits 7 to the final patient visit will be held at 4-monthly intervals.

(Note a 'month' is defined as a calendar month.)

Abbreviations

CRP	C-Reactive Protein
CTU	Clinical Trials Unit
ECG	Electrocardiogram
eGFR	Estimated Glomerular Filtration Rate
IMP	Investigational Medicinal Product
IV	Intravenous
LPLV	Last patient last visit
LVEF	Left Ventricular Ejection Fraction
MCH	Mean Cell Haemoglobin
MCHC	Mean Cell Haemoglobin Concentration
MCV	Mean Corpuscular Volume
N-BNP	N-terminal pro B-type Natriuretic Peptide
RDW	Red blood cell Distribution Width
TSAT	Transferrin saturation
