# Supplementary material for: Rationale and design of a randomised trial of intravenous iron in patients with heart failure
Source: Heart. 2022 Aug 10;108(24):1979–85. doi: 10.1136/heartjnl-2022-321304 (PMC9726969; doi:10.1136/heartjnl-2022-321304)
Supplement: Supplementary data [file heartjnl-2022-321304supp002.pdf]

Appendix 2:

Subgroup Analyses

Categorical variables:

- Sex (male or female)
- Recruitment in hospital, recent discharges, stable outpatients with raised BNP or NTproBNP
- Patients taking/not taking hypoglycaemic therapy
- TSAT <20% versus ferritin <100ug/L with TSAT ≥20%
- Aetiology of heart failure. (ischaemic vs non-ischaemic)
- NYHA (II vs III/IV)
- Duration of heart failure (new/<=1 year/>1 year)
- eGFR <=60 vs >60 (calculated by CKD-EPI)
- WHO anaemia definition (non-anaemic ≥12.0g/dL women/≥13.0g/dL men, mild – 11.0-11.9g/dL women /11.0-12.9g/dL men, moderate – 8.0-10.9g/dL)

Continuously distributed variables by thirds of their distributions of baseline:

- TSAT
- Ferritin
- Haemoglobin (after adding 1g/dL to the levels for women)
- Age
- Estimated glomerular filtration rate (eGFR, calculated by CKD-EPI)
- Systolic blood pressure
- Left ventricular ejection fraction

Powering of secondary endpoints

For hospitalisation for worsening heart failure there will be 80% power to detect a hazard ratio of 0.71 assuming at least 268 first events.

For cardiovascular hospitalisation there will be 80% power to detect a hazard ratio of 0.76 assuming at least 417 first events.

For cardiovascular death or heart failure hospitalisation there will be 80% power to detect a hazard ratio of 0.75 assuming at least 379 first events.

For the Minnesota Living with Heart Failure total score at 4 months there will be at least 80% power to detect a difference in mean scores of 4.5 assuming a common standard deviation of 24 and at least 450 subjects in each group with data.

Additional statistical methods and considerations

The randomisation schedule was based on randomised permuted blocks of size 4 and 6 in equal numbers nested within the stratification variables (recruitment context [hospital inpatient, recent discharge, and stable outpatient] and study site).

The proportional hazard assumption will be assessed by testing for significance the interaction of log(time) and treatment allocation in the model fitted.

Minnesota Living with Heart Failure scores and 6-minute walk test results will be compared between randomised treatment groups at 4 and 20 months using ANCOVA, with treatment group and stratification variable as covariates. For patients recruited as stable outpatients, these analyses will be repeated adjusting for baseline levels, in those patients whose baseline data are available. These analyses will be repeated using a multiple imputation procedure to account for missing data post-baseline. Missing values will be imputed within each treatment group separately using SAS PROC MI adjusting for the stratification variable. For the stable outpatient analysis, missing values will be imputed adjusting for the baseline value and stratification variable. Fifty datasets will be generated and results analysed by ANCOVA within each dataset and results combined using Rubin's rules using the SAS PROC MIANALYZE procedure.

In addition, EQ5D visual analogue scores and EQ5D indices will be summarised at each timepoint assessed with changes from baseline. For each of EQ5D visual analogue score and EQ5D index, a mixed effects repeated measures model with a general covariance matrix and including treatment main effect, study visit and stratification variable, will be used to estimate the average treatment effect over time, including data up to 3 years. In a second analysis, a heterogeneous treatment effect over time will be investigated by adding a treatment by study visit interaction to the model.

In addition to estimating treatment effects using cause specific Cox regression models as our primary analyses, we will also fit Fine and Gray analyses as sensitivity analyses.

The data are managed in a Microsoft SQL Server database according to the Glasgow CTU's standard operating procedures. All activities are monitored within our ISO 9001:2015 our Quality Management System and ISO/IEC 27001:2013 Information Security Management System, with regular external audits conducted by the British Standards Institute.

All statistical analyses will be carried out in SAS for Windows v 9.4, or higher and R version 3.6.0 or higher.
