# Supplementary material for: Rationale and design of a randomised trial of intravenous iron in patients with heart failure
Source: Heart. 2022 Aug 10;108(24):1979–85. doi: 10.1136/heartjnl-2022-321304 (PMC9726969; doi:10.1136/heartjnl-2022-321304)
Supplement: Supplementary data [file heartjnl-2022-321304supp003.pdf]

Appendix 3: Committee Membership

<u>Trial Steering Committee</u>

Dr Nicholas Boon (Chair)	Independent Member
Dr Shannon Amoils	British Heart Foundation, non-voting
Dr Callum Chapman	Co-applicant
Prof John Cleland	Co-applicant
Dr Thomas Goldin Diness	Pharmacosmos, non-voting
Prof Ian Ford	Study Director
Prof Paul Kalra	Chief Investigator
Prof Philip Kalra	Co-applicant
Prof Iain Macdougall	Co-applicant
Prof John McMurray	Independent Member
Mr Richard Mindham	Independent Member, Patient representative
Prof Mark Petrie	Co-applicant
Dr Pamela Sandu	Sponsor Representative, non-voting
Prof Iain Squire	Co-applicant
Dr Claes Christian Strom	Pharmacosmos, non-voting (past member)
Ms Elizabeth Thomson	Project Manager, non-voting
Dr Maureen Travers	Sponsor Representative, non-voting (past member)
Prof Robert Wilcox	Independent Member

<u>Independent Data Monitoring Committee</u>

Prof Allan Struthers (Chair)

Prof Patrick Mark

Prof Chris Weir

<u>Clinical Endpoint Committee</u>

Prof John Cleland (Chair)

Dr Fraser Graham

Dr Pierpaolo Pellicori

Trial Management Group

Ms Elizabeth Thomson (Chair)	Senior Project Manager
Ms Claire Brunton	Project Manager
Prof John Cleland	Clinical Endpoint Committee Chair
Ms Anna Cunnington	Research Coordinator (Portsmouth)
Ms Lisa Hall	Administrative Assistant (past member)
Dr Elizabeth Douglas	Senior Clinical Trials Pharmacist
Dr Margaret Fegen	Academic Clinical Trial Monitor
Prof Ian Ford	Study Director
Ms Serena Howe	Senior Research Nurse
Dr Marc Jones	Pharmacovigilance Manager
Prof Paul Kalra	Chief Investigator
Ms Sharon Kean	Director of Information Systems (past member)
Dr Sheila McGowan	Academic Lead Clinical Trial Monitor
Ms Jill Nicholls	Heart Failure Specialist Nurse
Prof Mark Petrie	Scottish Site Coordinator
Dr Barbara Ross	Academic Clinical Trial Monitor (past member)
Dr Pamela Sandu	Sponsor Research Coordinator
Dr Debra Stuart	Head of Research Regulation and Compliance
Ms Pamela Surtees	Specialist Pharmacy Technician - Clinical Trials
Dr Maureen Travers	Sponsor Research Coordinator (past member)

Trial Coordination

Portsmouth: Professor Paul Kalra (Chief Investigator), Anna Cunnington (Research Coordinator)

Robertson Centre for Biostatistics: Elizabeth Thomson (Project Management), Professor Ian Ford (Study Director), Michele Robertson and Nicola Greenlaw (Biostatistics), Sharon Kean (Information systems), Eleanor Dinnett (End points and clinical coding), Ross Clarke, Christopher Graham, Alan Stevenson and Robbie Wilson (eCRF), Sarah Boyle (Data Management), Lisa Hall and Joanne Woollard (Projects Administration), Claire Brunton and Amanda Reid (Endpoints Coordination)
