# Supplementary material for: Rationale and design of a randomised trial of intravenous iron in patients with heart failure
Source: Heart. 2022 Aug 10;108(24):1979–85. doi: 10.1136/heartjnl-2022-321304 (PMC9726969; doi:10.1136/heartjnl-2022-321304)
Supplement: Supplementary data [file heartjnl-2022-321304supp004.pdf]

Appendix 4: Principal Investigators of sites who randomised at least one patient to the IRONMAN trial

| | |
|---|---|
| Prof Paul Kalra | Queen Alexandra Hospital |
| Prof Mark Petrie | Glasgow Royal Infirmary and Golden Jubilee National Hospital |
| Dr Ninian Lang | Queen Elizabeth University Hospital |
| Prof Philip Kalra | Salford Royal Hospital |
| Dr Jason Glover | Basingstoke and North Hampshire Hospital |
| Prof Iain Squire | Glenfield Hospital |
| Dr Sue Ellery | Royal Sussex County Hospital |
| Dr Gethin Ellis | Royal Glamorgan Hospital |
| Dr Callum Chapman | West Middlesex University Hospital |
| Dr Fozia Ahmed | Manchester Royal Infirmary |
| Dr Ben Szwejkowski & Prof Chim Lang | Ninewells Hospital |
| Dr Clare Murphy | Royal Alexandra Hospital |
| Dr Charlotte Manisty | St Bartholomew's Hospital and University College Hospital |
| Prof Andrew Clark | Castle Hill Hospital |
| Prof Stephen Leslie | Raigmore Hospital |
| Dr Colin Petrie | University Hospital Monklands |
| Dr Alan Japp | Royal Infirmary of Edinburgh |
| Dr Jay Wright | Liverpool Heart and Chest Hospital |
| Dr Chris Critoph | Royal Bournemouth Hospital |
| Dr Andrew Hannah | Aberdeen Royal Infirmary |
| Dr Andrew Ludman | Royal Devon and Exeter Hospital |
| Prof Prithwish Banerjee | University Hospitals Coventry and Warwickshire |
| Dr Susan Piper | King's College Hospital |
| Dr Geraint Jenkins | Morriston Hospital |
| Dr Victor Chong | University Hospital Crosshouse |
| Dr Simon Williams | Wythenshawe Hospital |
| Dr Rebecca Lane | Royal Brompton and Harefield Hospital |
| Dr Patrick Donnelly | Ulster Hospital |
| Dr Paul Foley | Great Western Hospital |
| Dr Andrew Marshall | District General Hospital |
| Dr Amal Muthamala | North Middlesex University Hospital |
| Dr Philip Campbell | Royal Gwent Hospital |
| Dr Rajiv Sankaranarayanan | Aintree University Hospital |
| Dr Preeti Gupta & Dr Victor Sim | University Hospital Llandough |
| Dr Peter Cowburn | University Hospital Southampton |
| Dr Ameet Bakhai | Barnet Hospital |
| Dr Kristopher Lyons | Antrim Area Hospital |
| Dr Alison Seed | Blackpool Victoria Hospital |
| Dr Sudantha Bulugahapitiya | Bradford Royal Infirmary |
| Dr Justin Cooke | Chesterfield Royal Hospital |
| Prof Jerry Murphy | Darlington Memorial Hospital |
| Dr Catherine Labinjoh | Forth Valley Royal Hospital |
| Dr Piers Clifford | Hammersmith Hospital and Wycombe Hospital |
| Dr Charles Spencer | New Cross Hospital |

| | |
|---|---|
| Dr John Walsh | City Hospital |
| Dr Christopher Boos | Poole Hospital |
| Dr Abdallah Al-Mohammad | Northern General Hospital |
| Dr Thuraia Nageh | Southend University Hospital |
| Dr Mark Francis | Victoria Hospital |
| Dr Iain Matthews | Wansbeck General Hospital |
| Dr Sam McClure & Dr John Baxter | Sunderland Royal Hospital |
| Dr Philip Keeling | Torbay Hospital |
| Dr Lana Dixon | Royal Victoria Hospital |
| Dr Rebekah Schiff | Guy’s and St Thomas’ Hospital |
| Dr Ahmed Elzayat & Dr Alastair Cooke | Doncaster Royal Infirmary |
| Dr Simon Duckett | Royal Stoke University Hospital |
| Dr Robin Ray | St George’s Hospital |
| Dr Reto Gamma | Broomfield Hospital |
| Dr Eleanor Wicks | John Radcliffe Hospital |
| Dr Thomas Jackson | Salisbury District Hospital |
| Dr Tapesh Pakrashi | Kingston Hospital |
| Dr Jolanta Sobolewska | Royal Oldham Hospital |
| Dr Henry Savage | Basildon University Hospital |
| Dr Yuk-ki Wong | St Richard’s Hospital |
| Dr Aaron Wong | Princess of Wales Hospital |
| Dr Angus Nightingale | Bristol Royal Infirmary |
| Dr Parminder Chaggar | Royal Cornwall Hospital |
