# Supplementary material for: Rationale and design of a randomised trial of intravenous iron in patients with heart failure
Source: Heart. 2022 Aug 10;108(24):1979–85. doi: 10.1136/heartjnl-2022-321304 (PMC9726969; doi:10.1136/heartjnl-2022-321304)
Supplement: Supplementary data [file heartjnl-2022-321304supp005.pdf]

Appendix 5

Differences in trial design between IRONMAN, HEART-FID and FAIR-HF2

IRONMAN: Effectiveness of *I*n*tr*avenous ir*on* treat*m*ent vs standard care in p*a*tie*n*ts with heart failure and iron deficiency trial

HEART-FID trial: Ferric Carboxymaltose in Heart Failure With Iron Deficiency[1]

FAIR-HF2: Intravenous Iron in Patients With Systolic Heart Failure and Iron Deficiency to Improve Morbidity & Mortality[2]

Population

IRONMAN
New or established symptomatic heart failure with reduced ejection fraction (HFrEF): left ventricular ejection fraction (LVEF) ≤45% within the preceding 24 months
AND current or recent (within 6 months) hospitalisation for heart failure or elevated natriuretic peptide
Definition of iron deficiency: ferritin <100 µg/L or, provided ferritin ≤400 µg/L, TSAT<20%

HEART-FID
HFrEF with LVEF ≤40% within 24 months or ≤30% within 36 months
AND a documented hospitalisation for heart failure within 12 months or elevated NTproBNP within 90 days
Definition of iron deficiency: ferritin <100 µg/L, or between 100 and 300 µg/L if TSAT<20%

Estimated enrolment: 3014 participants

FAIR-HF2

HFrEF present for at least 12 months (no other data available)

Definition of iron deficiency: ferritin <100 µg/L, or between 100 and 300 µg/L if TSAT<20%

Estimated enrolment: 1200 participants

Trial design

IRONMAN: prospective, randomised open-label, blinded endpoint (PROBE) event-driven

HEART-FID: double-blind, placebo controlled

FAIR-HF2: double-blind, placebo controlled

Intravenous iron

IRONMAN: ferric derismaltose

HEART-FID: ferric carboxymaltose

FAIR-HF2: ferric carboxymaltose

Primary endpoint

IRONMAN: Combined rate of recurrent hospitalisations for heart failure and cardiovascular death

HEART-FID: A hierarchical scale of clinical severity comprising (i) death at 12 months, (ii) number of hospitalisations for heart failure at 12 months, or (iii) change in 6-minute walk test distance from baseline to 6 months.

FAIR-HF2: Combined rate of recurrent hospitalisations for heart failure and cardiovascular death

Intravenous (IV) iron re-dosing criteria

IRONMAN: Initial correction at baseline with infusion of IV ferric derisomaltose (for dosing see Table 2, additional dosing at 4 weeks if necessary). Every four months, IV ferric derisomaltose administered if either ferritin <100 µg/L or, provided ferritin ≤400 µg/L, TSAT <25%.

HEART-FID: Initial IV ferric carboxymaltose 750 mg followed by second 750 mg at 7 days (for patients >50kg). Re-dosing every 6 months in the IV ferric carboxymaltose arm according to haemoglobin (<13.5 g/dL in women and <15.0 g/dL in men) and iron status (ferritin <100 ng/mL or 100–300 ng/mL with TSAT<20%)

FAIR-HF2: Initial IV ferric carboxymaltose 1000 mg, followed by optional 500-1000 mg within the first 4 weeks. Subsequent administration of 500 mg IV ferric carboxymaltose every 4 months, except when haemoglobin >16.0 g/dL or ferritin >800 µg/L

[1] Mentz RJ, Ambrosy AP, Ezekowitz JA, et al. Randomized Placebo-Controlled Trial of Ferric Carboxymaltose in Heart Failure With Iron Deficiency: Rationale and Design. Circ Heart Fail. 2021;14(5):e008100.

[2] https://clinicaltrials.gov/ct2/show/NCT03036462 (accessed 4th June 2022)
